# Adoption and Usage of mHealth Technology on Quality and Experience of Care Provided by Frontline Workers: Observations From Rural India

**DOI:** 10.2196/mhealth.4047

**Published:** 2015-05-28

**Authors:** Sangya Kaphle, Sharad Chaturvedi, Indrajit Chaudhuri, Ram Krishnan, Neal Lesh

**Affiliations:** ^1^Dimagi Software InnovationsNairobiKenya; ^2^CARE IndiaPatnaIndia; ^3^Dimagi Software InnovationsCambridge, MAUnited States

**Keywords:** mHealth, technology adoption, community health workers, CommCare

## Abstract

**Background:**

mHealth apps are deployed with the aim of improving access, quality, and experience of health care. It is possible that any mHealth intervention can yield differential impacts for different types of users. Mediating and determining factors, including personal and socioeconomic factors, affect technology adoption, the way health workers leverage and use the technology, and subsequently the quality and experience of care they provide.

**Objective:**

To develop a framework to assess whether mHealth platforms affect the quality and experience of care provided by frontline workers, and whether these effects on quality and experience are different depending on the level of technology adoption and individual characteristics of the health worker. Literacy, education, age, and previous mobile experience are identified as individual factors that affect technology adoption and use, as well as factors that affect the quality and experience of care directly and via the technology.

**Methods:**

Formative research was conducted with 15 community health workers (CHWs) using CommCare, an mHealth app for maternal and newborn care, in Bihar, India. CHWs were first classified on the level of CommCare adoption using data from CommCareHQ and were then shadowed on home visits to evaluate their levels of technology proficiency, and the quality and experience of care provided. Regression techniques were employed to test the relationships. Out of all the CHWs, 2 of them refused to participate in the home visits, however, we did have information on their levels of technology adoption and background characteristics, which were included in the analysis as relevant.

**Results:**

Level of technology adoption was important for both quality and experience of care. The quality score for high users of CommCare was higher by 33.4% (*P*=.04), on average, compared to low users of CommCare. Those who scored higher on CommCare proficiency also provided significantly higher quality and experience of care, where an additional point in CommCare proficiency score increased the quality score by around half a point (0.541, *P*=.07), and experience score by around a third of a point (0.308, *P*=.03). Age affected CommCare user type negatively, with an increase in age increasing the likelihood of belonging to a lower category of CommCare adoption (-0.105, *P*=.08). Other individual characteristics did not affect adoption or the predicted values estimating the relationship between adoption and quality and experience of care, although illiteracy was able to affect the relationship negatively.

**Conclusions:**

mHealth technology adoption by frontline workers can positively impact the quality and experience of care they provide. Individual characteristics, especially literacy and age, can be important elements affecting technology adoption and the way users leverage the technology for their work. Our formative study provides informed hypotheses and methods for further research.

## Introduction

### Background

Mobile health, or mHealth, platforms are currently in use in various programs around the world to facilitate health care delivery where frontline health workers play an important role in providing health services in resource-poor settings. While the number of programs using mobile technologies in health care is increasing globally, there exists a significant gap in knowledge regarding its impact on health outcomes, as well as intermediary factors like access, quality, and experience [1,2].

Increased access, quality, and experience of care are known to contribute toward improved health outcomes. Traditionally, the impact of any health intervention is assessed by investigating changes in the relevant health outcomes that the intervention is targeting. Improvements in quality, access, or experience are rarely the focus of study, and how and whether the intervention is leading to improvements in these indicators is usually not investigated in detail.

Regardless of their impact on health outcomes, improvements in quality and experience of care are legitimate goals for any health intervention. Not only are they the intermediate outcomes for better health from an equity perspective, they can be seen as end goals of health interventions, whereby everyone has access to a high quality and experience of health care [3]. Mobile health interventions can target all three of these factors—access, quality, and experience—leading to improvements in health outcomes [2].

It is possible that any mHealth intervention can yield differential impacts for different types of users. The quality and experience of care provided by community health workers (CHWs) can depend on individual characteristics like literacy, education, or age; social factors like the perception of CHWs by their target communities; or health system factors like receiving adequate support and essential materials needed to do their job [4]. In the case of mHealth, novel individual factors can also come into play, including those that affect technology adoption, such as perception of technology relevance and self-efficacy in utilizing the tools [5]. Mediating and determining factors include personal and socioeconomic factors affecting technology adoption [5], the way the CHWs leverage and use the technology, and subsequently the quality and experience of care they provide. Additionally, adoption and usage of the technology can directly impact the quality and experience of care provided by CHWs, while personal factors can also have a direct impact on the quality and experience of care.

With this in mind, Dimagi, in collaboration with CARE India, conducted formative research in Bihar, India, to better understand how the use of mHealth platforms affect the quality and experience of care provided by different types of CHWs. Our aim is to develop a framework to analyze the effects of mHealth technology adoption on the quality and experience of care, and to provide greater understanding of the personal factors affecting (1) mHealth technology adoption and (2) CHWs’ use of the technology to support their work.

### Research Objective

Our objective is to develop a framework to assess whether mHealth platforms affect the quality and experience of maternal and newborn care provided by CHWs, and whether these effects on quality and experience are different depending on the level of technology adoption and individual characteristics of the CHWs. We identified literacy, education, age, and previous mobile technology experience as individual factors that affect technology adoption and use, as well as factors that affect the quality and experience of care via the technology. Literacy and education were also identified as potential factors which can directly affect the quality and experience of care provided. Our formative work also offers some insight into factors that may affect technology adoption, and provides some field observations for comparisons between quality and experience of care provided by those using and not using the technology.

We expect to find better quality and experience of care among CHWs with higher levels of adoption and usage of mHealth tools. We also expect different effects on quality and experience based on individual characteristics, in addition to the level of technology adoption and usage.

### The Setting

The study was conducted in Saharsa district in Bihar, India. Bihar is one of the more underdeveloped states in India. Most socioeconomic and mother-and-child health indicators in Bihar are considerably lower than the national average, including per-capita income, public expenditure on health, literacy rate, immunization rate among pregnant women and newborn children, institutional delivery rate, and malnutrition among children [6]. Nearly half of the population in Bihar is living under the poverty line [6]. Table 1 presents data on some socioeconomic and health indicators, as well as health infrastructure and human resource availability in Saharsa and Bihar. The low level of socioeconomic indicators adversely affects the health status of, and utilization of health services by, the population [6].

**Table 1:** Socioeconomic, health, and health infrastructure indicators for Saharsa and Bihar.

Indicators		Saharsa, n or %	Bihar, n or %
**Socioeconomic indicators**			
	Total literacy [7], %	53.20	61.80
	Male literacy, %	63.56	71.20
	Female literacy, %	41.68	46.40
	Total population [7], n	1,900,661	104,099,452
	Urban population, %	8.24	11.29
	Rural population, %	91.76	88.71
**Health indicators**			
	Crude birth rate (number of live births in reference period/mid-year population x 1000) [8]	31.2	26.1
	Crude death rate (number of deaths in reference period/mid-year population x 1000) [8]	7.4	6.8
	Infant mortality rate (number of infant deaths [less than 1 year of age]/number of live births during reference period x 1000) [8]	55	48
	Neonatal mortality rate (number of infants dying before 29 days per 1000 live births) [8]	37	32
	Postneonatal mortality rate (infants dying between 29 days and 1 year per 1000 live births) [8]	18	16
	Under 5 mortality rate (per 1000 live births) [8]	82	70
	Maternal mortality ratio (maternal deaths per 1000 live births) [8]	33	30
	Institutional deliveries, %	33.5 [9]	22.0 [10]
	Full immunization in children, %	52.4 [9]	39.8 [11]
**Health infrastructure and human resources [10]**			
	Number of doctors, n	53	N/A^a^
	Number of Auxiliary Nurse Midwives (ANMs), n	225	N/A
	Number of c (ASHAs), n	1242	N/A
	Number of Aganwadi Workers (AWWs), n	1367	N/A
	District hospitals, n	1	N/A
	Referral hospitals, n	0	N/A
	Primary health centers (PHCs), n	10	N/A
	Additional primary health centers (APHCs), n	15	N/A
	Health sub-centers (HSCs), n	152	N/A
	Blood banks, n	1	N/A

^a^Not applicable (N/A). The data for Bihar were unavailable.

**Figure 1:**
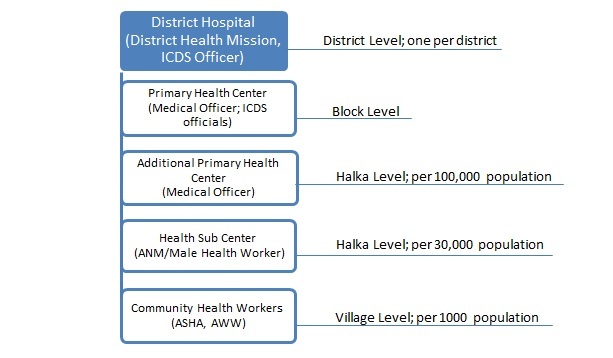
Public health system structure in India. The manpower available at each tier and administrative level of each type of health facility are shown. Each tier acts as a referral unit for the tier below. The district hospital services the facilities below with necessary support, resources, and essentials. Halka is a collection of villages, and Block is a collection of Halka.

Figure 1 describes the health system structure in place in Bihar’s districts including Saharsa. Each state in India has its own health care delivery system. The backbone of the system is a three-tier delivery system comprised of a tier one health sub-center (HSC), a tier two primary health center (PHC) and community health center (CHC), and a tier three district hospital. The Integrated Child Development Services (ICDS) provides immunization, health checkups, referrals, nutrition, health education, and preschool education to children below 6 years of age and women of reproductive age at the village level. Aganwadi Workers (AWWs) are at the center of the ICDS. AWWs run an Aganwadi center in each village, where the ICDS services are available to the population. However, the availability of health infrastructure and resources is still inadequate and the quality of services is poor. In order to improve the quality and access to services, especially in rural areas, the Ministry of Health and Family Welfare introduced the National Rural Health Mission (NRHM).

India announced and started implementing the NRHM with the goal of improving public health outcomes through community-driven approaches in 2005 [12]. The NRHM aims to improve access, affordability, accountability, and effectiveness of health care facilities available to the poor and vulnerable segments of the population. As part of the mission to bridge the gap in rural health services, the mission has created a cadre of community health workers, or Auxiliary Nurse Midwives (ASHAs), who are tasked with providing maternal and child health services to the communities that they are a part of. The ASHA program is a cornerstone of the NRHM, and it involves selecting, training, and supporting a locally recruited community-based health worker and change agent for every 1000 individuals in the community. The primary role of ASHAs is to create awareness and behavior change in health practices and improve utilization and accountability of the existing health systems, leading to stronger primary health care systems and services. ASHAs are trained to provide basic care, health information, and guidance, and to make referrals when appropriate. Community-led initiatives have been strikingly successful in improving health outcomes, and actions taken by households and families can prevent over 30% of child deaths [12].

CHWs, like ASHAs, have a vital role to play when it comes to influencing household and family choices that contribute to better health. In Bihar, where literacy levels are low, a majority of the ASHAs are from poor and low-literacy backgrounds [12]. ASHA training and support often involves content that requires more than basic literacy to grasp, which affects their effectiveness and performance. Working in remote, isolated settings, they can suffer from low morale and motivation. Capacity building using innovative training techniques suited for low-literacy adult learning, and equipping ASHAs with the resources to impart the knowledge, such as pictorial materials, radio access, or mHealth platforms, are some interesting strategies which are currently being deployed or being tested for their effectiveness to improve ASHA knowledge and skills [12]. ASHAs are complimented by AWWs, both of whom are supervised by an Auxiliary Nurse Midwife (ANM). The ANM runs village-level camps for maternal and child health services, which includes immunization of mothers and children, tracking nutritional status and growth, providing antenatal and postnatal checkups, and making referrals to the facilities when necessary [13].

There are some important challenges that need to be addressed in the health sector in Bihar. There are substantial gaps in health infrastructure, including primary health centers and community health centers. There are also gaps in the essentials required for effective functioning of the health facilities, including in drugs, consumables, equipment, and manpower [12]. Immunization coverage is low, there are high levels of malnutrition in children and mothers, and fertility rates are high [12]. Low quality of care provided at the district level down to the community levels, perpetuated by a lack of technical knowledge and skills, as well as gaps in health infrastructure and essentials, is also an important gap that needs to be addressed. Research on the challenges faced by ASHAs has identified a lack of support from PHC staff, a lack of adequate training, unclear incentives policy, and poor clarity in how to collaborate with the ANM and AWW as the main barriers to improving the quality of services they provide [14].

CARE India, in collaboration with the Ministry of Health and Ministry of Social Welfare, with the support of the Bill and Melinda Gates Foundation, is implementing CommCare, an mHealth platform targeting maternal and newborn care, with 600 ASHAs and AWWs in four blocks of the Saharsa district in Bihar. The deployment is part of a randomized controlled trial (RCT), which is implemented by CARE India with other consortium partners, and which will be evaluated by Mathematica Policy Research. The RCT compares health outcomes in different catchment areas where CHWs are (1) using CommCare or (2) using paper-based job aids. While our study is not associated with the broader RCT, the design of the intervention provides an ideal setting to better understand how mHealth platforms affect the quality and experience of home visits by CHWs. Both groups of ASHAs received content and capacity-building training facilitated by CARE at a fixed platform through an ANM, which means that CommCare ASHAs did not systematically receive more supervision and support than non-CommCare ASHAs. Additionally, the ASHAs using CommCare were randomly selected from the four blocks, which means that there was no bias to consider during the selection of our sample, which was drawn from the RCT’s sample of ASHAs using CommCare. We are able to observe differences in the performances of CHW’s using mHealth and those not using the technology and be more certain that any differences in visit quality and experience can be attributed to CommCare.

### Ethical Considerations

We have taken all measures possible to ensure that our study follows research governance and ethical protocol necessary for such research. The study benefits the participating ASHAs and the larger community where they work by understanding whether the mHealth platforms they are already using improve the quality and experience of the health services they provide to their communities. All ASHAs were informed about the nature of the study, and their consent was required prior to the researchers accompanying them on any home visits. Since our research does not involve patients or patient outcomes, it did not require any approvals from the Internal Review Board (IRB). Conflicts of interest that could arise due to the researchers having been employed by either Dimagi, who makes CommCare, or CARE India, who is implementing CommCare in Saharsa, have been mitigated by taking an unbiased view of CommCare in the study.

### Analytical Framework

#### mHealth Technology Adoption and Improvements in Quality and Experience of Care

The level of technology adoption and usage can significantly affect the quality and experience of care provided by CHWs using mHealth platforms. CHWs are using CommCare to record and track pregnancies, newborns, and children up to 2 years of age. CHWs use CommCare to register pregnancies, provide counseling to the mother and families on safe pregnancies and newborn care, register births and deaths, and track immunization histories for both mother and child. CommCare provides decision support; a reproductive health checklist to ensure comprehensive care; multimedia, including images, audio, and video to enhance behavioral change communication; and features to aid with work planning and scheduling. Data from CommCare is reported to a central database, which helps supervisors provide targeted supervision to the CHW. These features aid the CHW in providing better access, quality, and experience of care to her clients [2,15] by addressing the gaps in medical information and skills [1], increasing adherence to protocol and guidelines [16], and improving engagement of the client with the rich multimedia components of the app to help with behavior change [17].

#### Factors Influencing Technology Adoption

Rogers’ Diffusion Model identifies different users that adopt technology at various stages [18]. Other models of technology adoption identify demographic and individual characteristics, such as gender, age, technology advancement, technology readiness, technology experience, and self-efficacy, as mediating factors that affect technology adoption [5,19-21]. Demographic, socioeconomic, and individual factors can affect deterministic factors that affect technology adoption [5]. Perceived ease of use and perceived usefulness of technology are seen to affect attitudes and behaviors that influence the adoption and use of technology [5,20,21]. Social and program factors are also deemed important, but these are not the focus of our study [19,22,23].

CHWs are at various stages of technology adoption in our setting. Some CHWs have fully adopted the technology, others have not adopted the technology at all, while the remaining CHWs have only adopted certain features of the app.

#### Individual Factors and Their Effects on Community Health Workers’ Utilization of the Technology and Quality and Experience of Care

We identified age, education, literacy, and previous mobile experience as individual factors that can affect the way CHWs leverage the technology. Literacy and education can also have a direct impact on the quality and experience of care provided by the CHWs, regardless of their influence on the CHWs’ ability to leverage the app effectively.

The Ministry of Health (MOH) had imposed a minimum education criterion of 8 years to be eligible to work as an ASHA. However, not all ASHAs are educated to this level. Utilizing technology like CommCare effectively places additional cognitive demands on the CHWs’ attention and abilities [24], and it is possible that levels of literacy and education are important factors affecting quality and experience of care provided by the CHWs [25].

Partners implementing CommCare often cite low literacy and education among CHWs as a major challenge. In order to register patients and understand counseling messages in CommCare, CHWs require some level of comfort with reading and entering text into the app. If the CHWs are not literate, it is anticipated that they will be slow, require additional supervision and support, or be unable to adopt CommCare to facilitate their work. The quality of care they provide may also be lower since both CommCare and ASHA content training require that CHWs be able to read and write. CHWs with more years of education are able to better grasp the health information provided during the training.

CommCare includes audio prompts, images, and video to facilitate usage by lower-literacy users. An ASHA who is unable to read is still able to play the audio messages in order to understand the questions and to input appropriate responses. As such, she may be able to provide higher quality and experience of care compared to those not using or utilizing the technology effectively.

While previous mobile experience and age are not deemed to impact the quality or experience of care directly, as in the case of literacy or education, they can influence self-efficacy and perceived relevance and usability of the technology, mediating technology adoption and usage [26-28]. The impact on quality or experience of care is via the CHW’s ability to use and leverage the app. To facilitate technology adoption, all ASHAs are trained in the use of mobile phones, as well as in the app and its content by CARE.

**Figure 2:**
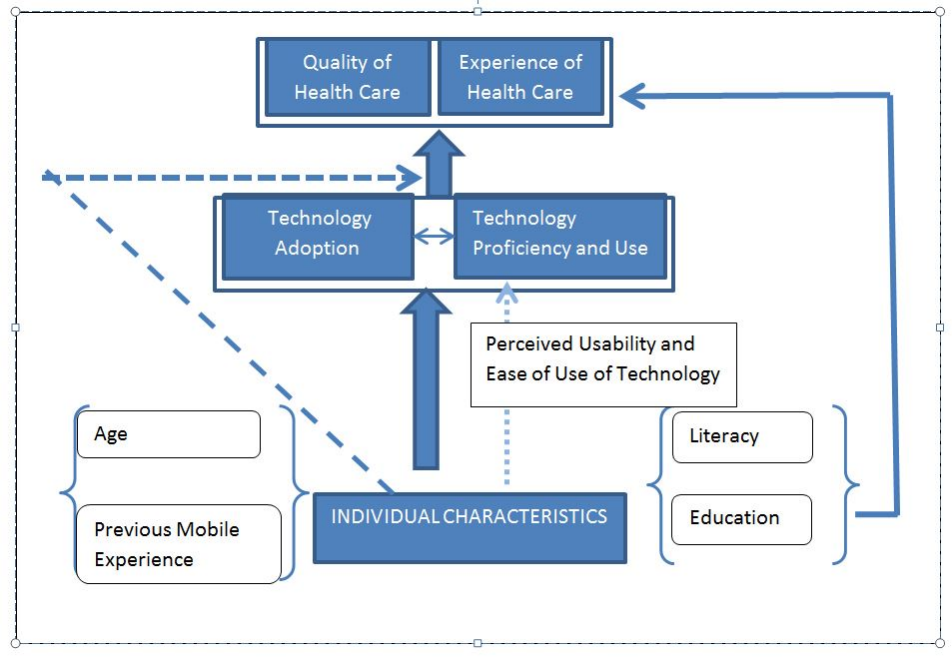
Analytical framework. The flowchart shows the relationship between mHealth technology adoption and usage, and quality and experience of care. The level of technology adoption can affect the quality and experience of care provided by CHWs because of the design and content of the app. Individual factors, including literacy, education, age, and previous mobile experience, are seen as mediating factors for mHealth technology adoption and usage. They influence the quality and experience of care by affecting the way CHWs leverage the technology to do their jobs. Literacy and education can also directly influence the quality and experience of care delivered by the CHW.

Figure 2 describes the analytical framework used in our study. This framework is adequate to study whether different levels of technology adoption and usage lead to differential effects of mHealth technology on quality and experience of care provided by the CHW. It is important to note that low adoption and usage of the technology, and low literacy and education, do not necessarily translate into low quality or experience of care. As long as the CHWs are able to leverage and adapt the technology to suit their skill set, they can provide high quality and experience of care even if they have low literacy and education levels, or are limited adopters and users of the technology. Social, cultural, and program factors are also important for mHealth technology adoption. Although these are not the focus of our study, our proposed model can benefit from including social, cultural, and program-wide factors, which also influence technology adoption and usage, and quality and experience of care.

## Methods

### Overview

We assessed the quality and experience of home visits of 13 ASHAs who are at different stages of CommCare adoption and use. We also assessed six home visits of 3 ASHAs who are not using CommCare, in order to better understand the effects of the mHealth platform on quality and experience of care. The ASHAs not using CommCare were selected based on availability and their proximity to CARE’s offices in Saharsa. We collected field observations from these visits to further inform our findings.

Tools to (1) assess home visit quality and experience for the client, and (2) measure CommCare proficiency were developed as part of the study. An additional survey was administered to all the ASHAs to collect information on their background characteristics, including literacy, education, age, and previous mobile experience. The assessment tools underwent iterations after two rounds of field testing. We then shadowed each ASHA on a home visit to observe and score her visit for quality and experience. We also shadowed 3 ASHAs who were not using CommCare, in order to collect observations on how home visits are conducted without the tool, and to note any differences in quality and experience of these visits.

### Data and Indicators

A total of 15 ASHAs were sampled and were classified as low (n=5), middle (n=5), and high (n=5) users of CommCare based on the number of forms submitted to CommCare’s central database in the past 90 days. Home visits of 14 ASHAs were observed to assess whether ASHA literacy, CommCare proficiency, and CommCare user type—low, middle, high—affected the quality and experience of care provided. There were 2 ASHAs, both low users of CommCare, who refused to participate in the home visits, although we were able to collect their background information. We had a total of 13 ASHAs in our sample, and we shadowed 1 ASHA, a low CommCare user, on two home visits, which means we had a total of 14 home visits for our analysis.

The specific indicators are discussed in Table 2. Continuous variables, including CommCare proficiency score, visit quality score, visit experience score, age, and previous mobile experience were classified as low, middle, or high in order to test for measures of association with the categorical variables literacy, education, observed visit quality, and CommCare user type. The number of form submissions in the last 30, 60, and 90 days was used to classify the type of CommCare user, which was used as a measure for technology adoption.

CommCare proficiency was measured directly during the home visit assessment as a composite score based on whether the ASHA could perform certain tasks in CommCare, and whether or not she used certain features of CommCare. We identified the following as features that measure CommCare proficiency and use: (1) navigation within the phone and within the app, (2) ability to select the client from a list of registered patients, (3) use of all the forms listed for the visit, (4) entering accurate answers, (5) entering text, dates, and numbers, and (6) the ability to play audio and video.

The quality of the home visit was measured through a quality score generated by scoring the visit based on (1) whether all the forms listed were used during the visit, (2) whether the visit included counseling, and (3) the number of counseling topics. For each topic, scoring was also based on (1) whether complete and accurate information was provided, (2) whether the client asked questions, and (3) whether the ASHA verified that the messages were received. Experience of home visit was measured directly during the home visit assessment. It is a composite score, generated by adding scores regarding (1) audio usage frequency, (2) video usage frequency, (3) frequency of showing images, (4) whether all of the people present were addressed, (5) whether the ASHA spoke clearly and loudly with confidence, and (6) the duration of the visit, where a visit under 10 minutes scored 1, 10 to 20 minutes scored 2, and over 20 minutes scored 3. The rationale for measuring visit experience in this way is based on the finding that the use of multimedia increases the quality of the visit [17].

Previous mobile experience was scored by asking whether AHSAs were able to perform a set of tasks on the mobile phone prior to using CommCare. A composite score was based on whether the ASHA (1) used a mobile phone, (2) owned her own phone, and (3) whether she could answer the phone, place calls, use the contact list, send and receive short message service (SMS) messages, play music, take photographs, change the phone’s date and time, check her balance, and charge the phone’s battery prior to using CommCare.

**Table 2:** List of indicators and their descriptions.

Indicators		Descriptions
**CommCare user type**		
	Low	Users who have not submitted any forms using CommCare in the last 90 days. We selected the 5 ASHAs who submitted the least number of forms in the last 90 days for this sample.
	Middle	Users who fell into the 50th percentile in terms of forms submitted in the last 30, 60, and 90 days. Those who fell between the 50th-55th percentiles for form submissions in the last 30, 60, and 90 days were the preferred middle users. In our sample, 3 users fell into this range for all three time periods and 2 fell into this range in the last 30 and 90 days.
	High	Users with the highest number of form submissions in the last 30, 60, and 90 days. In our sample, 3 users had the highest number of form submissions in all three time periods, and 2 ASHAs had the highest number in the last 30 and 60 days.
**CommCare proficiency** ^a^		
	Low	ASHAs in the lowest 25th percentile of the CommCare proficiency score were categorized as low.
	Middle	ASHAs within the 25th to 75th percentile of the CommCare proficiency score were categorized as middle.
	High	ASHAs above the 75th percentile of the CommCare proficiency score were categorized as high.
**Quality of home visit** ^b^		
	Low	ASHAs in the lowest 25th percentile of the visit quality score were categorized as low.
	Middle	ASHAs within the 25th to 75th percentile of the visit quality score were categorized as middle.
	High	ASHAs above the 75th percentile of the visit quality score were categorized as high.
Observed visit quality		A second measure for visit quality based on the researcher’s perception of the home visit was included. This was a subjective measure of the visit quality, classified again as low, middle, or high, based on the researcher’s perception.
**Experience of home visit** ^c^		
	Low	ASHAs in the lowest 25th percentile of the visit experience score were categorized as low.
	Middle	ASHAs within the 25th to 75th percentile of the visit experience score were categorized as middle.
	High	ASHAs above the 75th percentile of the visit experience score were categorized as high.
**Literacy level** ^d^		
	Illiterate	The ASHA cannot read at all.
	Low Literacy	The ASHA can read with difficulty, or can read some of the sentence.
	Literate	The ASHA can read easily.
**Education level** ^e^		
	Low	The ASHA was educated up to 8th standard.
	Middle	The ASHA was educated up to 10th standard.
	High	The ASHA was educated up to, or higher than, 12th standard.
Previous mobile experience^f^		Previous mobile experience was classified as low, middle, or high based on the percentile, where those under the 25th percentile score were low, 25th-75th percentile were middle, and above the 75th percentile were high.
Age^g^		Age was classified as low, middle, or high based on the percentile, where 25th percentile and below were low, 25th-75th percentile were middle, and above 75th percentile were high.

^a^ASHAs could earn a maximum of 22 points for their CommCare proficiency score.

^b^ASHAs could receive a maximum of 22 points for their quality of home visit score.

^c^ASHAs could receive a maximum of 16 points for their visit experience score.

^d^A literacy test was administered as part of the background interviews to assess the literacy levels, where ASHAs were asked to read a sentence in Hindi out loud.

^e^Education was self-reported by the ASHA during the interview.

^f^ASHAs could score a maximum of 18 points for previous mobile experience.

^g^Age was self-reported by the ASHA during the interview.

### Limitations of the Indicators

The tool to measure the visit quality and experience had not been rigorously tested prior to the formative work, and our measure of the visit quality and experience has room for improvement. Currently, the quality score is mostly dependent on the number of counseling topics, where if an ASHA counsels on three topics she will have a larger possible score even if the counseling is not necessarily of high quality, than if she counsels on one topic. Although it is likely to be more comprehensive, it is not necessarily the case that a larger number of counseling topics equals higher-quality visits. The scoring system for quality and experience of visit can also be changed so that most important aspects are weighted accordingly. Another possibility is to assess the components of quality and experience independently, without creating a composite quality or experience score. Along this line, it may also be beneficial to create a single score for both quality and experience combining the different elements into one single indicator. Despite the shortcomings, we are confident that the tool captures the quality and experience of the home visit without bias.

### Empirical Strategy

The empirical strategy adopted below will be supplemented by field observations to understand four specific elements of our analytical model.

#### 1. Does mHealth Technology Adoption Affect Quality and Experience of Care?

We will start by using both of the indicators of adoption, CommCare proficiency and usage, as well as CommCare user type based on form submissions to understand the effects of technology adoption on quality and experience of care. We will use the quality and experience scores as our dependent variables since the observed visit quality measure is more subjective.

We first estimate how CommCare adoption measured by CommCare user type affects the quality and experience of care provided by the ASHA using a simple linear regression model.

Quality_ij_ = β0+β1COMMCAREuser-type1_ij_ +β2COMMCAREuser-type3_ij_+ϵ_ij_ (1.1)

Experience_ij_ = β0+β1COMMCAREuser-type1_ij_ +β2COMMCAREuser-type 3_ij_+ϵ_ij_ (1.2)

We estimate two different specifications for equations 1.1 and 1.2 and include two out of the three different categories of CommCare user type in each specification, which allows us to compare all three groups of users against each other. The coefficient for β1 and β2 estimate the difference in quality and experience score for that CommCare user type against the CommCare user type excluded from the specification.

We then estimate specifications 1.3 and 1.4 using CommCare proficiency as the indicator for CommCare adoption. We again use a linear regression model to estimate specifications 1.3 and 1.4. β1 estimates how a 1-point increase in CommCare proficiency affects quality/experience of care.

Quality_ij_ = β0+β1COMMCAREProficiency_ij_ +ϵ_ij_ (1.3)

Experience_ij_ = β0+β1COMMCAREProficiency_ij_ +ϵ_ij_ (1.4)

#### 2. Do Accredited Social Health Activist Characteristics Affect Adoption of CommCare?

We aim to see the effects of ASHA characteristics, namely literacy levels, education level, age, and previous mobile experience, on CommCare adoption and usage by estimating the following two models. Model 2.1 estimates how the ASHA characteristics affect their categorization of CommCare user type using an ordered probit model, while model 2.2 estimates how these characteristics affect their CommCare proficiency using a multivariate regression model. For education and literacy, we create binary variables. Since our sample for illiterate is small, we estimate specification 2.1 with illiterate and low literacy users combined into one group, and those with low and middle education levels combined into one group, and compare results against literate and highly educated users. We also follow this method for specification 2.2.

CommCare user-type_ij_ = β0+β1Lit_ij_+β2Edu_ij_+β3Age_ij_ +β4PrevMobileExp_ij_+ϵ_ij_ (2.1)

CommCareproficiency_ij_ = β0+β1Lit_ij_+β2Edu_ij_+β3Age_ij_ +β4PrevMobileExp_ij_+ϵ_ij_ (2.2)

We also estimated the relationship for all three categories of education, without combining the low- and middle-educated users into one category. However, this did not change our results.

#### 3. Do Accredited Social Health Activist Characteristics, Namely Literacy and Education, Affect Quality and Experience of Care Directly?

We focus on literacy and education since age and previous mobile experience likely do not have any direct association with quality and experience of care. We estimate specifications 3.1 and 3.2 using a linear regression model. We estimate 3.1 and 3.2 with the illiterate and literate users combined into one group, and those with low and middle education levels combined into one group, and compare results against literate and highly educated users due to the small sample of illiterate users.

Quality_ij_=β0+β1Lit_ij_ +β3Edu_ij_ +ϵ_ij_ (3.1)

Experience_ij_=β0+β1Lit_ij_+β2Edu_ij_ +ϵ_ij_ (3.2)

We also estimated the relationship for all three categories of education, without combining the low- and middle-educated users into one category. However, this did not change our results and they are not presented in this paper.

#### 4. Do Accredited Social Health Activist Characteristics Matter for the Relationship Between CommCare Adoption and Quality and Experience of Care Identified in the First Model (1.1-1.4)?

ASHA characteristics can affect their ability to leverage the technology. We estimate how ASHA characteristics affect the relationship between CommCare adoption and quality and experience of care by using the predicted values for quality score and experience score from equations 1.1 and 1.2 as our dependent variables. These predicted values estimate the relationship between quality/experience of care and CommCare user type, a measure for adoption in our study. Using these predicted values as our dependent variables will allow us to gauge whether the relationship between quality/experience of care and technology adoption is affected by individual characteristics.

Using predicted values from equations 1.1 and 1.2, respectively, we estimate models 4.1 and 4.2 treating these as a system of equations. The dependent variables are predicted values from models 1.1 and 1.2, which estimate the relationship between quality/experience of care and CommCare user type.

Quality'_ij_ = β0+β1Lit_ij_+β2Edu_ij_+β3Age_ij_ +β4PrevMobileExp_ij_+ϵ_ij_ (4.1)

Experience'_ij_ = β0+β1Lit_ij_+β2Edu_j_+β3Age_ij_ +β4PrevMobileExp_ij_+ϵ_ij_ (4.2)

Using predicted values from equations 1.3 and 1.4, respectively, we estimate equations 4.3 and 4.4. The dependent variables are predicted values from equations 1.3 and 1.4, which estimate the relationship between quality/experience of care and CommCare proficiency, a second measure for technology adoption in our study.

Quality'_ij_ = β0+β1Lit_ij_+β2Edu_ij_+β3Age_ij_ +β4PrevMobileExp_ij_+ϵ_ij_ (4.3)

Experience'_ij_ = β0+β1Lit_ij_+β2Edu_ij_+β3Age_ij_ +β4PrevMobileExp_ij_+ϵ_ij_ (4.4)

## Results

### Descriptive Statistics

Table 3 presents the descriptive statistics of the individual ASHA characteristics informing the study, and Table 4 presents the descriptive statistics for the ASHAs' home visit quality and experience scores for different levels of CommCare adoption. CommCare user type was positively and significantly correlated with CommCare proficiency, with a correlation coefficient of .771 (*P*=.001), significant at the 99% confidence level. There was also significant positive correlation between our two measures of quality with a correlation coefficient of .787 (*P*<.001), also significant at the 99% confidence level.

**Table 3:** Descriptive statistics of ASHA characteristics.

ASHA characteristics		Mean (SD) or n (%)
Age in years (n=15), mean (SD)		31.60 (5.86)
Previous mobile experience (n=15), mean (SD)		8.25 (4.23)
CommCare proficiency and use (n=14), mean (SD)		8.78 (4.84)
**Education (n=15), n (%)**		
	Low, n (%)	5 (33)
	Middle, n (%)	4 (27)
	High, n (%)	6 (40)
**Literacy (n=15), n (%)**		
	Illiterate, n (%)	1 (7)
	Low Literacy, n (%)	6 (40)
	Literate, n (%)	8 (53)
**CommCare user type (n=15), n (%)**		
	Low, n (%)	5 (33)
	Middle, n (%)	5 (33)
	High, n (%)	5 (33)
**CommCare proficiency (n=14), n (%)**		
	Low, n (%)	3 (21)
	Middle, n (%)	7 (50)
	High, n (%)	4 (29)

**Table 4:** Descriptive statistics for quality/experience of home visits for different levels of CommCare adoption.

ASHA CommCare adoption (n=14)		n (%)	Quality score, mean (SD)	Experience score, mean (SD)	Perception of visit quality			
					Mean (SD)	Low, n (%)	Middle, n (%)	High, n (%)
CommCare visits (n=14)		14 (100)	7.92 (5.24)	5.57 (2.59)	2.00 (0.88)	5 (36)	4 (29)	5 (36)
Non-CommCare visits (n=6)		6 (100)	9.17 (4.75)	4.33 (1.37)	1.50 (0.84)			
**CommCare** **user type**								
	Low	4 (29)	4.25 (0.50)	3.25 (0.96)	1.00 (0)	4 (29)		
	Middle	5 (36)	7.20 (6.38)	5.80 (2.39)	2.00 (0.71)	1 (7)	3 (21)	1 (7)
	High	5 (36)	11.60 (4.16)	7.20 (2.59)	2.80 (0.45)		1 (7)	4 (29)
**CommCare proficiency**								
	Low	3 (21)	4.33 (0.58)	3.67 (0.58)	1.00 (0)	3 (21)		
	Middle	7 (50)	8.00 (6.22)	5.71 (3.14)	2.00 (0.82)	2 (14)	3 (21)	2 (14)
	High	4 (29)	10.50 (4.43)	6.75 (1.89)	2.75 (0.50)		1 (7)	3 (21)

The mean age of the ASHAs was 31.60 years (SD 5.86) and most self-reported having 8 years of education.  Out of the 15 ASHAs, only 1 (7%) was classified as illiterate in the sample, while 6 (40%) were low literacy, and 8 (53%) were literate. The mean visit quality score for ASHAs using CommCare was 7.92 (SD 5.24), while the mean experience score was 5.57 (SD 2.59).  We only had 3 ASHAs that were not using CommCare included in the study, and we observed two home visits for each of these 3 ASHAs for a total of six home visits. Their visit quality and experience scores were modified versions of the CommCare visit quality and experience scores, excluding the mobile technology components. As such, the quality and experience scores are not entirely comparable across the two groups of ASHAs. However, we can look at the score for perception of visit quality as an unbiased indicator capturing the quality of the visit for both CommCare and non-CommCare ASHAs. Based on this indicator, the perception of visit quality was higher for ASHAs using CommCare compared to those not using CommCare. Similarly, mean quality and experience scores for ASHAs with higher levels of CommCare adoption were higher compared to those with lower levels of CommCare adoption using both measures of adoption.  

ASHAs who were literate had a higher mean for the experience score compared to those that had low literacy or were illiterate, as seen in Table 5. However, means for quality and experience did not seem to increase along with increases in the levels of education, literacy, and previous mobile experience. Older ASHAs had lower visit quality and experience means compared to their younger compatriots.

**Table 5:** Quality and experience scores by ASHA individual characteristics.

ASHA characteristics (n=14)		n (%)	Quality score, mean (SD)	Experience score, mean (SD)	Perception of visit quality			
					Mean (SD)	Low, n (%)	Middle, n (%)	High, n (%)
**Literacy**								
	Illiterate	2 (14)	4.00 (0)	3.50 (0.71)	1.00 (0)	2 (14)		
	Low literacy	6 (43)	8.67 (6.53)	5.67 (3.44)	2.00 (0.89)	2 (14)	2 (14)	2 (14)
	Literate	6 (43)	8.50 (4.64)	6.17 (1.83)	2.33 (0.82)	1 (7)	2 (14)	3 (21)
**Education in years**								
	8	6 (43)	7.33 (6.25)	5.50 (2.51)	1.83 (0.98)	3 (21)	1 (7)	2 (14)
	10	3 (21)	5.67 (2.89)	3.67 (2.08)	1.67 (0.58)	1 (7)	2 (14)	
	≥12	5 (36)	10.00 (5.15)	6.80 (2.68)	2.40 (0.89)	1 (7)	1 (7)	3 (21)
**Previous mobile experience**								
	Low	5 (36)	7.00 (5.61)	5.40 (2.30)	2.00 (0.71)	1 (7)	3 (21)	1 (7)
	Middle	6 (43)	9.17 (5.95)	6.33 (3.01)	2.17 (0.98)	2 (14)	1 (7)	3 (21)
	High	3 (21)	7.00 (4.36)	4.33 (2.51)	1.67 (1.15)	2 (14)		1 (7)
**Age**								
	Low	3 (21)	6.00 (5.20)	5.67 (2.31)	2.00 (1.00)	1 (7)	1 (7)	1 (7)
	Middle	7 (50)	10.43 (5.80)	6.86 (2.61)	2.43 (0.79)	1 (7)	2 (14)	4 (29)
	High	4 (29)	5.00 (2.00)	3.25 (0.96)	1.25 (0.50)	3 (21)	1 (7)	

### Correlation Coefficients and Regression Results

#### 1. Does the Level of mHealth Technology Adoption Affect Quality and Experience of Care Provided by Accredited Social Health Activists?

Table 6 presents the correlation coefficients between CommCare adoption and quality and experience of health care. While CommCare user type is significantly associated only with perception of the visit quality, CommCare proficiency and use is significantly correlated with all three response variables: quality score, experience score, and perception of visit quality.

**Table 6:** Correlations between CommCare adoption and quality and experience of care.

Variables^a^	χ^2^ or *r* ^b^	*P*
Quality score and CommCare user type, χ^2^_2_	4.6	.33
Experience score and CommCare user type, χ^2^_2_	2.5	.65
Perception of visit quality and CommCare user type, χ^2^_4_	14.3	.006
Quality score and CommCare proficiency, *r*	.50	.07
Experience score and CommCare proficiency, *r*	.57	.03
Perception of visit quality and CommCare proficiency, χ^2^_2_	9.3	.06

^a^We transformed quality and experience scores into categorical variables in order to test for association with CommCare user type, which is also a categorical variable.

^b^We performed chi-square tests to look for measures of association between categorical variables, and pairwise correlations (*r*) for continuous variables.

We found that the level of technology adoption is important for both quality and experience of care. High users of CommCare, as identified by CommCare user type, provided significantly higher scores for quality and experience of care than low users of CommCare for both measures of quality. The quality score for high users of CommCare was higher by 7.35 (*P*=.04), on average, compared to low users of CommCare, which is a difference of 33.4%, significant at the 95% confidence level. Those who scored higher on CommCare proficiency also provided significantly higher quality and experience of care scores, where an additional point in the CommCare proficiency score increased the quality score by around half a point (0.54, *P*=.07), and experience score by around a third of a point (0.31, *P*=.03). This amounts to a 2.5% increase in quality score, and a 1.9% increase in experience score for each additional point in CommCare proficiency, both significant at the 95% confidence level (see Table 7).

**Table 7:** Relationship between CommCare adoption and quality and experience of care.

ASHA characteristics (n=14)		Quality score, mean (*t*_13_)		Experience score, mean (*t*_13_)			Quality score^a^, mean (*t*_13_)		Experience score^b^, mean (*t*_13_)
		1^c^	2	3	4		5		6
**CommCare user type**									
	Low	-2.950 (-0.96)		-2.550 (-1.74)					
	Middle		2.950 (0.96)			2.550 (1.74)			
	High	4.400 (1.51)	7.350 (2.38)	1.400 (1.01)		3.950 (2.70)			
CommCare proficiency								0.541 (2.00)	0.308 (2.43)
Constant^d^		7.200 (3.50)	4.250 (1.85)	5.800 (5.94)		3.250 (2.98)		3.172 (1.18)	2.866 (2.28)

^a^The increase in quality score as a result of a 1-point increase in proficiency.

^b^The increase in experience score as a result of a 1-point increase in proficiency.

^c^The numbers 1 to 6 in this row represent the specifications that were run for the model.

^d^The constant is the value for β0 in our model, or when all the variables are estimated at 0.

#### 2. Do Individual Characteristics Matter for mHealth Technology Adoption and Usage?

Age is the only factor that was correlated with CommCare proficiency and usage as seen in Table 8. When we combined illiterate and low literacy users and compared with literate users, we did not find any significant differences in CommCare adoption using both measures.

Age affected CommCare user type negatively, with an increase in age increasing the likelihood of belonging to a lower category of CommCare user type (-0.105, *P*=.08). Age also affected the CommCare proficiency score negatively, with each additional year decreasing the CommCare proficiency score by 0.4 points (*P*=.09), but only when low and middle levels of literacy and education were combined into one variable. Based on these results, we can hypothesize that although illiteracy could influence adoption, in general, lower literacy, education, and previous mobile experience do not affect CommCare adoption, while age can be an influencing factor for adoption.

Further analysis, not presented here, compared CommCare proficiency and usage scores for low-literacy ASHAs and the illiterate ASHA. The illiterate ASHA had lower CommCare proficiency and usage scores, with illiteracy decreasing the CommCare proficiency score by 41% compared to lower literacy. Compared to literate ASHAs, the illiterate ASHA had a CommCare proficiency score that was 51% lower. Both these results were significant at the 95% confidence level. However, as we only had one illiterate ASHA in our sample, who we had observed during multiple home visits, we cannot treat this single ASHA as a category and these results cannot be the basis to conclude that illiteracy affects CommCare proficiency and usage. Hence, these results are not presented in this paper. Table 9 shows the relationship between CommCare adoption and ASHA characteristics.

**Table 8:** Correlations between CommCare adoption and ASHA characteristics.

Variables^a^	χ^2^ or *r* ^b^	*P*
CommCare user type and age, χ^2^_2_	4.8	.31
CommCare user type and literacy^c^, *F*		.54
CommCare user type and education, χ^2^_2_	2.5	.65
CommCare user type and previous mobile experience, χ^2^_2_	6.2	.19
CommCare proficiency and age, *r*	-.5137	.06
CommCare proficiency and previous mobile experience, *r*	-.2700	.35
CommCare proficiency and literacy, *F*		.001
CommCare proficiency and education, χ^2^_2_	6.3	.18

^a^We transformed continuous variables, age and previous mobile experience, into categorical variables in order to test for association with CommCare user type, which is also a categorical variable.

^b^We performed chi-square tests to look for measures of association between categorical variables, and pairwise correlations (*r*) for continuous variables.

^c^We used Fisher’s exact test (*F*) for literacy since we only have one observation for illiteracy.

**Table 9:** Relationship between CommCare adoption and ASHA characteristics.

ASHA characteristics	Specification 1		Specification 2	
	CommCare user type^a^ (n=16), mean (*t*_15_)	*P*	CommCare proficiency^b^ (n=14), mean (*t*_13_)	*P*
Illiterate plus low literacy	0.127 (0.17)	.87	-1.963 (-0.67)	.52
Education (low plus middle)	-0.969 (-1.21)	.23	-2.616 (-0.85)	.42
Previous mobile experience	-0.0968 (-1.24)	.21	-0.139 (-0.47)	.65
Age	-0.105 (-1.77)	.08	-0.402 (-1.90)	.09
_cut1^c^	-5.093 (-2.32)	N/A^d^		
_cut2^c^	-4.055 (-1.92)	N/A		
Constant^e^			25.86 (3.71)	.005

^a^The ordered probit model was applied for this analysis.

^b^Ordinary least-squares (OLS) regression was used for generalized linear modelling.

^c^_cut1 and _cut2 are ancillary parameters and do not have associated *P* values. The coefficients show the estimates for the cutoff points chosen by the model for our categorical dependent variable.

^d^Not applicable (N/A).

^e^The constant is the value for β0 in our model, or when all the variables are estimated at 0.

#### 3. Do Levels of Literacy and Education Affect Quality and Experience of Care Directly?

We did not find any association between literacy levels and quality and experience of care, or education levels and quality and experience of care provided by the ASHAs. Table 10 shows the correlations between the quality and experience of care and ASHA characteristics. The results from Table 11 estimating effects of literacy and education on quality and experience of care also did not show any significant effects of literacy or education on quality and experience of care.

**Table 10:** Correlations between quality and experience of care and ASHA characteristics.

Correlated variables	χ^2a^	*P*
Quality and literacy^b^, *F*		.32
Experience and literacy, *F*		.14
Observed quality and literacy, *F*		.48
Quality and education, χ^2^_2_	7.22	.13
Experience and education, χ^2^_2_	5.33	.26
Observed quality and education, χ^2^_2_	4.55	.34

^a^Chi-square tests were used to look for measures of association between categorical variables.

^b^Fisher’s exact test (*F*) was used for literacy since we only have one observation for illiteracy.

**Table 11:** The effect of literacy and education levels on quality and experience of care.

ASHA characteristics	Specification 1	Specification 2
	Quality score^a^ (n=14), mean (*t*_13_)	Experience score^a^ (n=14), mean (*t*_13_)
Illiterate plus low literacy	1.085 (0.31)	-0.010 (-0.01)
Education (low plus middle)	-3.849 (-1.06)	-1.906 (-1.08)
Constant^b^	9.783 (3.89)	6.802 (5.57)

^a^Ordinary least-squares (OLS) regression was used for generalized linear modelling.

^b^The constant is the value for β0 in our model, or when all the variables are estimated at 0.

#### 4. Do Accredited Social Health Activist Characteristics Matter for the Relationship Between CommCare Adoption and Quality and Experience of Care?

Most ASHA characteristics also did not seem to affect the relationship between CommCare adoption and quality and experience of care. Literacy level did not seem to affect the quality of the visit by leveraging the way that ASHAs are able to use the technology, and had no direct impact on quality or experience of care. Combining illiterate and low literacy users together, we did not find any significant effect of literacy levels on the relationship between quality/experience and CommCare proficiency and CommCare user type (see Table 12).

However, other analysis (not presented here) showed that predicted values estimating the relationship between CommCare proficiency and quality of care were negatively affected  for the illiterate user compared to lower literacy users (-4.95, *P*=.09). Similarly,  predicted values estimating the relationship between CommCare proficiency and experience of care were also negatively affected for the illiterate user compared to lower literacy users (-2.815, *P*=.09). These findings indicate that illiteracy does seem to affect the relationship between CommCare proficiency and quality score, as well as the relationship between CommCare proficiency and experience of care. These results are not presented here due to the small sample size of illiterate ASHAs, and should be seen as an informed hypothesis for further study. Table 13 shows the effect of ASHA characteristics on the relationship between CommCare user type and the quality and experience of care.

Age also negatively affected CommCare proficiency and usage. Predicted values estimating the relationship between CommCare proficiency and quality/experience of care were both negatively affected, while there was no such effect for CommCare user type.

**Table 12:** The effect of ASHA characteristics on the relationship between CommCare proficiency and quality/experience of care.

ASHA characteristics	Specification 1		Specification 2	
	Quality’^a^ (n=14), mean (*t*_13_)	*P*	Experience’^a^ (n=14), mean (*t*_13_)	*P*
Illiterate plus low literacy	-1.063 (-0.67)	.52	-0.605 (-0.67)	.52
Education (low plus middle)	-1.417 (-0.85)	.42	-0.806 (-0.85)	.42
Age	-0.218 (-1.90)	.09	-0.124 (-1.90)	.09
Previous mobile experience	-0.0751 (-0.47)	.65	-0.0427 (-0.47)	.65
Constant^b^	17.17 (4.55)	.001	10.83 (5.05)	.001

^a^The dependent variable is the predicted value from the first model estimating the relationship between CommCare proficiency and quality and experience of care.

^b^The constant is the value for β0 in our model, or when all the variables are estimated at 0.

**Table 13:** The effect of ASHA characteristics on the relationship between CommCare user type and quality/experience of care.

ASHA characteristics	Specification 1		Specification 2	
	Quality’^a^ (n=14), mean (*t*_13_)	*P*	Experience’^a^ (n=14), mean (*t*_13_)	*P*
Illiterate plus low literacy	0.831 (0.42)	.69	0.227 (0.22)	.83
Education (low plus middle)	-2.270 (-1.12)	.29	-0.793 (-0.74)	.47
Age	-0.230 (-1.59)	.14	-0.113 (-1.50)	.16
Previous mobile experience	-0.143 (-0.70)	.50	-0.147 (-1.38)	.20
Constant^b^	17.02 (3.62)	.004	10.52 (4.25)	.001

^a^The dependent variable is the predicted value from the first model estimating the relationship between commcare proficiency and quality and experience of care.

^b^The constant is the value for β0 in our model, or when all the variables are estimated at 0.

### Field Observations

#### Observations of Non-CommCare Users

We observed six home visits of ASHAs who were not using CommCare. Most visits without CommCare were short and incomplete. The visits focused on the immediate state of the baby or mother, rather than assessing their health since the last visit, and the information provided was targeted only to the current situation of the mother or child. For example, during a visit with a mother and her newborn who had a cough/cold, an ASHA only counseled the mother to take the child to the doctor and did not address other aspects of newborn care. In another instance, we visited a day-old newborn. The ASHA passed on inaccurate information, wrapping up the child in a blanket and advising the mother to hold him against herself, enclosed in a blanket, for skin-to-skin contact. From these visits, we can suggest that CommCare increases the comprehensiveness of home visits, and decreases instances of inaccurate counseling**.**

#### Observations of CommCare Users: Stages of Adoption

Low and middle CommCare users tended to use CommCare as a reporting tool by filling out the checklists in the app without providing counseling or elaborating on the messages. For these users, the accuracy of their reporting can be brought into question as they can simply press “yes” for all questions without asking the client for a response. Reporting “yes” for everything would bring down an ASHA’s CommCare proficiency and visit quality scores, because certain questions, counseling prompts, and videos are only displayed based on the answers input for previous questions.

High users of CommCare seemed to understand the design and purpose of CommCare, and were able to move beyond using CommCare as a reporting tool. They tended to elaborate on the messages and provide more thorough counseling to the client. This may be because users first concentrated their efforts on learning how to use the app, and then moved on to grasping the content and design. ASHAs first focused on using CommCare as a reporting tool before using it as a job aid to support them during home visits, to plan their schedules, and provide targeted information to the clients. Using CommCare appropriately increased the quality of home visits, the accuracy of information passed on to the clients, and the experience of home visits for the client.

For the middle users, we believe that CommCare can play an interesting role in increasing the quality and experience of care. CommCare in Bihar had been deployed for around 6 months at the time of this study. The middle users were mostly focused on using CommCare as a reporting tool. At this stage, the multimedia in CommCare plays a key role in increasing the quality of the visit. Playing the audio and video and showing images means that the client is getting some information about a comprehensive range of topics, even if the ASHA is not providing any counseling.

If the low and middle users, initially providing low-quality care, do not use the media in CommCare, the quality of the home visit will remain poor, as the ASHA is entirely focused on filling out the checklist, sometimes without asking the client any questions. In this case, the quality of the home visit is much higher without CommCare, as in that case the ASHA would at least pass on some messages to the client, however incomplete.

The reasons we uncovered for low CommCare use are idiosyncratic, depending on attitude, training, literacy, and age/health. Of these, attitude and training seemed to be the most influential, and it is most likely that high users were providing a high level of care without CommCare, and the low users were providing a low quality of care without CommCare. Our observations showed us that the high users were more dynamic, confident, and capable in their roles as ASHAs, while low users were less dynamic and comfortable in their roles as ASHAs. However, their attitudes and dynamism did not seem to be significantly correlated with education or literacy. Although our research tools did not include any way to measure attitude, it is possible to discern the variety of attitudes present from the ASHAs’ behavior. For example, two ASHAs who were low users of CommCare refused to perform a home visit for the study, though they were literate and had higher levels of education.

## Discussion

### Limitations of the Study

Our formative study had some important limitations. First, our sample size was too small to establish conclusive evidence to describe the relationships we were testing. Second, we only studied differences between using mHealth tools and not using mHealth tools for quality and experience of care qualitatively via field observations. Third, social and program factors that can affect technology adoption and quality and experience of care directly and via the technology were not analyzed and were outside the scope of the study. Lastly, simply observing the ASHAs’ home visits could have had an effect on performance leading to biased indicators. Observing the ASHAs can potentially induce better performance due to the perception of a supervisor or outsider being present, or can induce worse performance due to a feeling of pressure or nervousness.

### Conclusions

Based on our findings, we identified two levels of CommCare adoption. The first level of adoption was where the ASHAs were still learning the design and content of the technology, and used it as a tool for reporting. The second level of adoption was where they were more proficient in using CommCare, understood how the tool is designed, and used it appropriately as a job aid for reporting, as well as for counseling during home visits.  ASHAs in the first stage of adoption had lower quality and experience of home visits, compared to those in the second stage of adoption. Though the causality from proficiency to adoption is not clear, and adoption affects proficiency and vice versa, it was demonstrated that higher proficiency leads to higher adoption, and to quality and experience of care. Individual characteristics, other than illiteracy, did not seem to affect proficiency nor adoption, and further research is required to reach concrete conclusions about the effects of illiteracy on proficiency and adoption of mHealth tools.

A higher level of CommCare adoption was significantly associated with higher quality and experience of care, although it is possible that these users were already providing higher quality and experience of care. While individual characteristics, including education and previous mobile experience, studied here did not affect the stages of adoption nor the quality or experience of home visits, illiteracy can affect the quality and experience of care by influencing CommCare adoption, as can the way ASHAs leverage the technology to provide care. Using multimedia effectively was more prominent in those that displayed higher levels of CommCare adoption. Low literacy users were still able to use mHealth technology to provide higher quality and experience of care, however, illiterate users do need more support and training to understand the design and workflow of mHealth apps, and to accrue the benefits of the technology.

The small sample size in our study means that our results should be taken as informed hypotheses for further study. The relationship between levels of mHealth technology adoption and quality and experience of care can be established with a larger sample size using the methods presented in this paper. Any further research should include a reliable sample of literate, lower-literacy, and illiterate users to test the model for mHealth technology adoption and quality and experience of care presented in this paper.

## Abbreviations

ANM: Auxiliary Nurse Midwife
APHC: additional primary health center
ASHA: Accredited Social Health Activist
AWW: Aganwadi Worker
CHC: community health center
CHW: community health worker
HSC: health sub-center
ICDS: Integrated Child Development Services
IRB: Internal Review Board
MOH: Ministry of Health
N/A: not applicable
NRHM: National Rural Health Mission
OLS: ordinary least squares
PHC: primary health center
RCT: randomized controlled trial
SMS: short message service
